# Vaccine willingness: Findings from the COVID-19 effects on the mental and physical health of Asian Americans & Pacific Islanders survey study (COMPASS)

**DOI:** 10.1016/j.pmedr.2021.101480

**Published:** 2021-07-09

**Authors:** Van M. Ta Park, Marcelle Dougan, Oanh L. Meyer, Bora Nam, Marian Tzuang, Linda G. Park, Quyen Vuong, Janice Y. Tsoh

**Affiliations:** a Department of Community Health Systems, School of Nursing, University of California, San Francisco, 2 Koret Way, San Francisco, CA, USA; b Asian American Research Center on Health (ARCH), University of California, San Francisco, San Francisco, CA, USA; c Department of Public Health and Recreation, San Jose State University, One Washington Square, San Jose, CA, USA; d Department of Neurology, University of California, Davis, Davis, CA, USA; e International Children Assistance Network, Milpitas, CA, USA; f Department of Psychiatry and Behavioral Sciences, School of Medicine, University of California, San Francisco, San Francisco, CA, USA

**Keywords:** COVID-19, Vaccine willingness, Asian Americans, Pacific Islanders

## Abstract

Willingness to get the COVID-19 vaccine is crucial to reduce the current strain on healthcare systems and increase herd immunity, but only 71% of the U.S. public said they would get the vaccine. It remains unclear whether Asian Americans and Pacific Islanders (AAPI), a population with existing inequalities in COVID-19 infection and mortality, are willing to get the vaccine, and the factors associated with vaccine willingness.

Given this imperative, we used data from a national, cross-sectional, community-based survey called **C**OVID-19 Effects **o**n the **M**ental and **P**hysical Health of **A**API **S**urvey **S**tudy (**COMPASS**), an ongoing survey study that is available in English and Asian languages (i.e., Simplified or Traditional Chinese, Korean, Vietnamese) to examine vaccine willingness among AAPI.

A total of 1,646 U.S. adult AAPI participants completed the survey. Self-reported vaccine willingness showed the proportion who were “unsure” or “probably/definitely no” to getting the COVID-19 vaccine was 25.4%. The odds for vaccine willingness were significantly lower for were Native Hawaiians and Pacific Islanders (vs. Asian Americans), Korean Americans (vs. Chinese and Vietnamese Americans), women (vs. men), heterosexuals (vs. non-heterosexuals), those aged 30–39 and 50–59 (vs. aged < 30), and those who reported having any vaccine concerns (vs. no concerns).

AAPIs’ willingness to get COVID-19 vaccine varied by groups, which underscores the need for disaggregated AAPI data. A multi-pronged approach in culturally appropriate and tailored health communication and education with AAPI is critical to achieve the goal of health equity for AAPI as it pertains to COVID-19 mortality and morbidity.

## 1 Introduction

As the number of Americans infected and dying from coronavirus disease 2019 (COVID-19) continues to increase (33,470,212 and 601,808 respectively, as of June 29, 2021 (Centers for Disease Control and Prevention, 2021), it is vital that the U.S. population receive the COVID-19 vaccines to effectively mitigate this pandemic. In December 2020, the Food and Drug Administration (FDA) authorized Pfizer and Moderna COVID-19 vaccines for emergency use. (Food and Administration, 2021, Food and Administration, 2021)On February 27, 2021, the FDA provided an emergency use authorization for Janssen COVID-19 vaccine. (Food and Administration, 2021)As of June 29, 2021, 381,831,830 COVID-19 vaccine doses had been distributed and 325,152,847 persons had received one or more doses in the U.S. (Centers for Disease Control and Prevention. COVID-19 Vaccinations in the United States., 2021)

Vaccine uptake is crucial to reduce the strain on healthcare systems and increase herd immunity, albeit the percent that must receive the vaccine to achieve herd immunity remains unknown. (World Health Organization. Coronavirus disease (COVID-19): Herd immunity, lockdowns and COVID-19., 2021)Surveys conducted in the early months of the pandemic revealed that 31–50% of Americans were hesitant about receiving the COVID-19 vaccine. (Fisher et al., 2020, Callaghan et al., 2020)A recent poll found 27% said they “probably/definitely would not” despite its safety or availability at no-cost. (Kaiser Family Foundation, 2020)Factors reportedly associated with vaccine hesitancy included being younger in age (Fisher et al., 2020), self-identification as Black (Fisher et al., 2020, Callaghan et al., 2020, Kaiser Family Foundation, 2020, Reiter et al., 2020), being a woman (Callaghan et al., 2020), lower educational attainment7, (Head et al., 2020), being an essential worker (Kaiser Family Foundation, 2020), and working in a health care setting9, (Head et al., 2020). Some reasons for vaccine hesitancy included vaccine-specific concerns (e.g., safety) (Fisher et al., 2020, Callaghan et al., 2020, Kaiser Family Foundation, 2020, Reiter et al., 2020), a need for more information (Fisher et al., 2020), antivaccine attitudes/beliefs7, (Kaiser Family Foundation, 2020), concerns about getting COVID-19 from the vaccine (Kaiser Family Foundation, 2020), and a lack of trust (Fisher et al., 2020).

There remains limited understanding about willingness to get the COVID-19 vaccineamong Asian Americans and Pacific Islanders (AAPI), the fastest growing U.S. racial population. (Pew Research Center. Key facts about Asian Americans, a diverse and growing population., 2021)While two surveys had found that Asian Americans (vs. Whites) are more likely to say they would get vaccinated (e.g., 56–83%% of Asians; 46–61% of Whites (Franklin Templeton-Gallup Economics of Recovery Study. Roundup of Recent Gallup Data on Vaccines., 2021, Pew Research Center. Intent to Get a COVID-19 Vaccine Rises to 60% as Confidence in Research and Development Process Increases., 2021), these surveys were limited to English-speaking Asian Americans and data were aggregated. AAPI are heterogenous in their histories, cultures, and languages (Pew Research Center. Key facts about Asian Americans, a diverse and growing population., 2021); and, having disaggregated data would be crucial in public health vaccine efforts to AAPI. The Revisions to the Standards for the Classification of Federal Data on Race and Ethnicity (OMB Statistical Policy Directive No. 15) define minimum federal standards for collecting and presenting data on race and ethnicity, including “Asian” and “Native Hawaiians or Pacific Islander (NHPI)” as separate racial groups. (Office of Management and Budget. Revisions to the Standards for the Classification of Federal Data on Race and Ethnicity., 2021)Additionally, research has shown that language accessibility has important implications for health, health care access, and research participation. (Chen et al., Nov 2007, Jacobs et al., 2006, Watson et al., Apr 2014) (Census Bureau, 2021) AAPI are also heterogenous in other aspects such as wealth/income. (Ishimatsu et al., 2021, Center for American Progress, 2021)For example, the median annual earnings for full-time, year-round women workers vary widely by AAPI subpopulation with Asian Indian, Malaysian, and Taiwanese earning $70,000 and Southeast Asian and NHPI earning about half or less. (Center for American Progress, 2021)AAPI are also heterogenous in health burdens. For example, AAPI groups were substantially different in the prevalence of obesity, smoking, and diabetes (e.g., Filipinos had the highest age-sex adjusted prevalence of diabetes among AAPI, but also greater than all other racial/ethnic groups). (Gordon et al., 2019)Aggregated data contributes to the “model minority” stereotypes (e.g., having economic success; no health problems), but the realities are clear with disaggregated data. (Ishimatsu et al., 2021, Gordon et al., 2019)

COVID-19 morbidity and mortality are not as well studied for AAPI (vs. other major racial/ethnic populations). Data suggest that AAPI, as a whole or specific groups, experience high rates of COVID-19 cases and deaths. Based on electronic health record data of 50 million patients from 53 health systems across 21 states, Asians (vs. White) had higher hospitalization (15.9 vs 7.4 per 10,000) and death rates (4.3 vs. 2.3 per 10,000) from COVID-19 as of July 2020. (Rubin-Miller et al., 2021)Based on the National Center for Health Statistics’ data on attributable death from COVID-19 from February-October 2020, AAPI had higher attributable COVID-19 deaths than Whites among age groups 45–84; NHPI also had higher attributable mortality among those aged 15–24. (Chu et al., 2021)In San Francisco, Asian Americans had a four times higher case fatality rate vs. the overall population (5.2% vs. 1.3%) a few months into the pandemic. (Yan et al., 2020)The sparse disaggregated data for AAPI revealed that some groups had disproportionate COVID-19-related burdens. For example, based on data from 85,328 patients who tested for COVID-19 at New York City’s public hospital system, South Asians had the highest rates of positivity and hospitalization among all Asians, which was only second to Hispanics for positivity and Blacks for hospitalization; and, Chinese patients had the highest mortality rate of all groups and were about 1.5 times more likely to die from COVID-19 (vs. Whites). (Marcello et al., 2020)

Without important data about COVID-19 vaccine willingness, it remains unclear whether health disparities for AAPI, a population with existing inequalities in COVID-19 infection and mortality, (Marcello et al., 2020) may be exacerbated. As such, this study examined the proportion and factors associated with COVID-19 vaccine willingness among AAPI using data from a national ongoing, multilingual survey, **C**OVID-19 Effects **o**n the **M**ental and **P**hysical Health of **A**API **S**urvey **S**tudy (**COMPASS**).

## 2 Methods

### 2.1 Study Eligibility, recruitment and procedures

COMPASS is a cross-sectional, community-based national survey that assesses the COVID-19 effects on AAPI. To be eligible, participants must self-identify as AAPI; be able to read English/Chinese/Korean/Vietnamese; be ≥ 18 years old; and reside in the U.S. The survey is available online (https://compass.ucsf.edu/), phone, and limited in-person. This paper reports on 1,646 participants who completed the survey from October 24-December 11, 2020, which was selected as the cutoff date for this analysis since it was the first day that the FDA authorized a COVID-19 vaccine. (Food and Administration, 2021)Mean survey completion time was 21.6 (standard deviation (SD) = 15.5) minutes. There were 100 incomplete surveys. Single persons (vs. married) were significantly less likely to be incomplete responders. There were no significant differences by other background/social characteristics or by vaccine willingness. Each participant was provided an option of receiving a $10 gift card upon survey completion.

Convenience sampling was employed in this study. Participants heard about COMPASS through organizations who serve AAPI, personal/professional networks, social media, email/listservs, flyers, and ethnic media. COMPASS also leveraged the Collaborative Approach for AAPI Research and Education (CARE) Registry (Collaborative Approach for Asian Americans and Pacific Islanders Research Education. CARE Registry for Asian Americans and Pacific Islanders., 2021) to recruit participants.

Participants provided e-informed consent (n = 1,535) or verbal phone consent (n = 111). The survey used the Research Electronic Data Capture hosted at the University of California, San Francisco (UCSF). (Harris et al., Apr 2009, Harris et al., 2019)The World Health Organization’s process of translation and adaptation of instruments (World Health Organization. Process of translation and adaptation of instruments., 2018) was used to guide the translations of the study materials that were not already available in the targeted language(s).

### 2.2 Measures

#### 2.2.1 Dependent variable

*Willingness to Get the COVID-19 Vaccine Item* was modified from another survey. (Neumann-Böhme et al., 2020)Participants were asked, “If a vaccine becomes available for COVID-19, would you get it?” Likert response options included: definitely yes; probably yes; unsure; probably no; and, definitely no.

#### 2.2.2 Independent variables

*Socio-demographic* items were drawn from CARE. (Collaborative Approach for Asian Americans and Pacific Islanders Research Education. CARE Registry for Asian Americans and Pacific Islanders., 2021)Variables included race (Asian; NHPI; American Indian/Alaskan Native; Black/African American; White/Caucasian), cultural group (Asian Indian/Chinese/Huaren/Filipino/Korean/Japanese/Native Hawaiian/Samoan/Taiwanese/Vietnamese/Other/Mixed), sex (Female/Male; Other/Prefer not to answer), sexual orientation (Heterosexual/Not heterosexual/Prefer not to answer), year of birth, nativity (country of birth), years lived in the U.S., marital status (Single; Married; Living with a partner; Separated/Divorced; Widowed), employment (Full-time/Part-time/Homemaker/Unemployed/Retired/Other), education (option ranged from grade to Graduate school), and household income (options ranged from ≤$25,000 to ≥$200,0001). Participants were asked how well they could speak/read/write English (very well/well/some/a little bit/not at all).

Participants completed several existing surveys related to COVID-19 including the *Coronavirus Impact Scale (CIS)* related to changes in family income/employment (School, 2020) and *COVID-19 status* (Cawthon et al., 2020) (yes, no, unsure diagnosis). *COVID-19 Vaccine Concerns* (Neumann-Böhme et al., 2020) included the following response options (check all that apply): 1) I do not have any concerns; 2) I'm concerned about potential side effects; 3) I think COVID-19 vaccine may not be safe; 4) I do not think that COVID-19 is dangerous to my health; 5) I am against vaccination in general; 6) The best way is for nature to take its course; 7) I believe natural or traditional remedies; 8) I'm afraid of injections; 9) Religious reasons; and, 10) Other.

Participants answered duration of *Shelter-in-Place (SIP)* questions based on *region*, per the Census Bureau’s definition of region (Midwest/Northeast/South/West), (Bureau, 2021)which was obtained by converting the zip code, or internet protocol address in the case of missing zip codes (n = 188). *SIP and Perceived Severity of COVID-19* items were developed by COMPASS.

*General Health* was measured by asking participants to indicate their health “today” on a scale from 0 (worst) to 100 (the best health you can imagine) using the EQ-5D (EuroQol, Dec 1990, Rabin and de Charro, Jul 2001) item, which was categorized into quintiles.

### 2.3 Statistical analysis

Chi-squared tests were used to examine the association between vaccine willingness, modeled as a 5-level categorical variable (definitely yes/probably yes/unsure/probably no/definitely no) and hypothesized factors associated with vaccine willingness, specifically race, cultural group, sex, sexual orientation, age, nativity, marital status, employment, education, household income, vaccine concerns, length of shelter in place, perceived severity of COVID-19, effect of coronavirus on family income, region, and general health status. Participants who responded that they could speak/read/write English less than very well (“some”, “a little bit”, or “not at all”) were categorized as having limited English proficiency (LEP). (Census Bureau, 2021)COVID-19 concerns were first classified as none, side-effects only, unsafe only, and multiple reasons for descriptive purposes. Then, it was further categorized as a binary variable (none or any concerns) in subsequent regression analyses to examine the association between vaccine willingness and any type of vaccine concerns.

This study used multinomial logistic regression to model the association between vaccine willingness, categorized as “definitely yes” (reference group), “probably yes”, “unsure/probably no/definitely no”, and these same factors. The “definitely yes” and “probably yes” responses differed conceptually. “Definitely” implied commitment whereas “probably” implied ambivalence and unwillingness to commit, which have differential associations with behavioral outcomes. (Baca-Motes et al., 2013, Rice et al., 2017)Consistent with previous research on vaccine willingness (Brandt et al., 2021, Ernesto, 2020)and guided by the data revealing differences in the two response groups in their proportion of participants with no vaccine concerns (44.1% vs, 9.7%, *p* < 0.01), the two responses were treated as separate outcome categories. The ‘unsure,’ ‘probably no,’ and ‘definitely no’ were combined into a single outcome category due to sample size considerations and the 3 groups appear to be similar in their associations with other factors. This study first conducted bivariate multinomial logistic regression to identify candidate variables for the final model. Since it was important not to miss variables that may become significant in the presence of others, this study retained variables that that attained a *p*-value of < 0.10 in bivariate analyses in the final modelLin et al., 2020–12–17 2020;14 (Zoran Bursac et al., 2008, Ranganathan et al., 2017). All statistical tests were two-sided. Hosmer-Lemeshow goodness of fit test (Hosmer and Lemesbow, 1980) indicated an acceptable fit of the final model (*p* = 0.27). The variables included in the final model were race, cultural group, sex, sexual orientation, age, nativity, marital status, employment status, income, vaccine concerns, and COVID severity. Statistical analyses were conducted using SAS Software. (SAS Institute Inc, 2014)

### 2.4 Human subjects protection

This study was approved by UCSF’s Institutional Review Board (protocol 20-31925).

## 3 Results

### 3.1 Sample Characteristics

The mean age of participants (N = 1,646) was 40.6 years (SD: 15.8) and ranged from 18 to 88. Participants included 62.6% female, 90.0% heterosexuals, and 97.6% Asian Americans. Major cultural groups included ethnic Chinese (including persons from Hong Kong and Taiwan; 37.1%), Vietnamese (29.0%), and Korean (20.5%). Overall, 61.5% of participants were foreign-born who had lived in the U.S. an average of 47.8% of their lives (SD: 24.0%), 20% spoke limited English, and 73.3%completed the survey in English (Table 1)

**Table 1:** Study Sample Characteristics (N = 1,646).

Characteristics	N	%
**Willingness to be vaccinated**		
Definitely yes	725	44.0
Probably yes	503	30.6
Unsure	295	17.9
Probably no	82	5.0
No	41	2.5
**Race**		
Asian	1,607	97.6
NHPI	39	2.4
**Cultural Group**		
Asian-Indian	28	1.7
Ethnic Chinese^1^	611	37.1
Filipino	71	4.3
Japanese	29	1.8
Korean	337	20.5
Native Hawaiian	17	1.0
Samoan	13	0.8
Vietnamese	477	29.0
Other/Mixed	63	6.9
**Sex**		
Female	1,028	62.5
Male	601	36.5
Other/Decline^2^	17	1.0
**Sex -orientation**		
Heterosexual	1,478	90.0
Not heterosexual	95	5.8
Decline to state	69	4.2
**Age**	40.6 (15.8)^3^; Range: 18 – 88	
<30	535	32.5
30–39	339	20.6
40–49	237	14.4
50–59	295	17.9
>60	240	14.6
**Nativity**		
US-born	619	37.6
Foreign-born	1,012	61.5
**Percent of life lived in the U.S.**	47.8 (24.0)^3^; Range: 0 – 100	
Don't know	15	0.9
**Limited English Proficiency (LEP)**^4^		
LEP in speaking	335	20.4
LEP in reading	309	18.8
LEP in writing	337	20.5
**Marital Status**		
Single	564	34.3
Married/Living with partner	988	60.0
Separated/Divorced/Widowed	83	5.0
Declined	11	0.7
**Employment Status**		
Full time	735	44.7
Part-time	287	17.4
Homemaker	150	9.1
Unemployed	213	12.9
Retired	141	8.6
Other/declined	120	7.3
**Education**		
High school or less	247	15.2
Some college or technical school	238	14.6
Bachelor's degree	633	39.0
Master's degree or higher	507	31.2
**Household Income ($)**		
≤ 25,000	283	17.2
> 25,000 –75,000	530	32.2
>75,000 – 150,000	408	24.8
> 150,000	258	15.7
Decline to state	167	10.2
**Tested positive for coronavirus**		
Yes	33	2.0
No	1485	90.2
Not sure	108	6.7
Missing	20	1.2
**Concerns about COVID-19 vaccines**		
None	386	23.5
Side effects only	562	34.1
Unsafe only	99	6.0
Multiple reasons	599	36.4
**Length of SIP**^5^**order**		
No order	93	5.7
< 1 month	111	6.8
1 to < 2 months	227	13.8
2 to < 3 months	223	13.6
3 months or longer	855	52.1
Don't know	132	8.0
**The severity of COVID where you live**		
A lot less	146	8.9
Somewhat less	338	20.6
About the same	417	25.4
Somewhat more	502	30.6
A lot more	236	14.4
**Effect on family income/employment**		
No change	587	35.9
Mild	554	33.9
Moderate	439	26.8
Severe	56	3.4
**Census Region**		
Midwest	90	5.8
Northeast	142	8.6
South	305	18.5
West	1109	67.4
**Self-reported HealthQuintiles (range of health score)**	78.2 (15.7)^3^; Range: 1–100	
Q1 (1 – 70)	345	22.2
Q2 (71 – 78)	264	17.0
Q3 (79 – 83)	324	20.8
Q4 (84 – 90)	351	22.6
Q5 (91 – 100)	270	17.4

1 Ethnic Chinese includes mainland Chinese, Hongkonger, Taiwanese, and Huaren.

2 Other: n = 7; Decline: n = 6

3 Mean (SD)

4 English proficiency categorized as limited if speak/read/write English indicated as “some”, “a little” or “not at all”

5 SIP: Shelter-in-Place

### 3.2 COVID-19 analyses

As shown in Table 1, 44.0% of participants said, “definitely yes”, 30.6% “probably yes”, 17.9% “unsure”, 5.0% “probably no”, and 2.5% “definitely no” to getting the COVID-19 vaccine. About 24% reported no vaccine concerns. One in three (34.1%) were only concerned about side effects, 6.0% were only concerned about vaccine safety, and 36.4% reported multiple concerns. >64% reported that the COVID-19 had impacted their family income and employment. Over half (52.1%) indicated their SIP was ≥ 3 months; 45.0% perceived the COVID-19 severity of where they lived was “somewhat” to “a lot” more than other areas. Few (2.0%) said they had tested positive for COVID-19, 90.2% had tested negative, and 7.9% who were unsure or did not provide a response.

### 3.3 Bivariate analyses

In bivariate analyses, race, cultural group, sex, sexual orientation, age, nativity, marital status, income, vaccine concerns, and COVID-19 severity were significantly associated with vaccine willingness, but LEP, employment status, education, length of SIP order, effect on family income/employment, region, and self-reported health status, and caregiver status were not (Table 2).

**Table 2:** Willingness to Be Vaccinated, by Participant Characteristics (N = 1,646).

Characteristics	Definitely yes	Probably yes	Unsure	Probably no	Definitely no	p-value
	N = 725	N = 503	N = 295	N = 82	N = 41	
**Race**						<0.001
Asian	715 (44.5)	493 (30.7)	288 (17.9)	76 (4.7)	35 (2.2)	
NHPI	10 (25.6)	10 (25.6)	7 (18.0)	6 (15.4)	6 (15.4)	
**Cultural Group**						<0.001
Ethnic Chinese	246 (40.3)	216 (35.4)	113 (18.5)	24 (3.9)	12 (2.0)	
Filipino	27 (38)	25 (35.2)	13 (18.3)	5 (7)	1 (1.4)	
Korean	114 (33.8)	120 (35.6)	62 (18.4)	26 (7.7)	15 (4.5)	
Vietnamese	273 (57.2)	105 (22)	76 (15.9)	19 (4)	4 (0.8)	
Other/Mixed	65 (43.3)	37 (24.7)	31 (20.7)	8 (5.3)	9 (6)	
**Sex**						<0.001
Female	430 (41.8)	304 (29.6)	216 (21.0)	49 (4.8)	29 (2.8)	
Male	288 (47.9)	195 (32.5)	76 (12.7)	32 (5.3)	10 (1.7)	
Other/Decline	7 (41.2)	4 (23.5)	3 (17.7)	1 (5.9)	2 (11.8))	
**Sexual Orientation**						0.002
Heterosexual	643 (43.5)	457 (30.9)	269 (18.2)	72 (4.9)	37 (2.5)	
Not heterosexual	56 (59.0)	26 (27.4)	6 (6.3)	7 (7.4)	0 (0)	
Decline to state	25 (36.2)	19 (27.5)	19 (27.5)	2 (2.9)	4 (5.8)	
**Age**						<0.001
<30	239 (44.7)	173 (32.3)	84 (15.7)	29 (5.4)	10 (1.9)	
30–39	120 (35.4)	125 (36.9)	67 (19.8)	17 (5.0)	10 (3.0)	
40–49	98 (41.4)	69 (29.1)	45 (19.0)	16 (6.8)	9 (3.8)	
50–59	124 (42.0)	82 (27.8)	63 (21.4)	17 (5.8)	9 (3.1)	
>60	144 (60.0)	54 (22.5)	36 (15.0)	3 (1.3)	3 (1.3)	
**Nativity**						0.007
Foreign-born	475 (46.9)	284 (28.1)	180 (17.8)	47 (4.6)	26 (2.6)	
US-born	246 (39.7)	212 (34.3)	113 (18.3)	35 (5.7)	13 (2.1)	
Don't know	4 (26.7)	7 (46.7)	2 (13.3)	0 (0)	2 (13.3)	
**LEP**^1^**in speaking**						0.622
Yes	155 (46.3)	93 (27.8)	65 (19.4)	14 (4.2)	8 (2.4)	
No	570 (43.5)	410 (31.3)	230 (17.5)	68 (5.2)	33 (2.5)	
**LEP**^1^**in reading**						0.879
Yes	141 (45.6)	87 (28.2)	58 (18.8)	16 (5.2)	7 (2.3)	
No	584 (43.7)	416 (31.1)	237 (17.7)	66 (4.9)	34 (2.5)	
**LEP**^1^**in writing**						0.929
Yes	149 (44.2)	99 (29.4)	64 (19)	18 (5.3)	7 (2.1)	
No	576 (44)	404 (30.9)	231 (17.7)	64 (4.9)	34 (2.6)	
**Marital Status**						0.095
Single	245 (43.4)	173 (30.7)	102 (18.1)	31 (5.5)	13 (2.3)	
Married/Living with partner	422 (42.7)	313 (31.7)	176 (17.8)	50 (5.1)	27 (2.7)	
Separated/Divorced/Widowed	52 (62.6)	13 (15.7)	16 (19.3)	1 (1.2)	1 (1.2)	
Declined	6 (54.5)	4 (35.4)	1 (9.1)	0 (0)	0 (0)	
**Employment Status**						0.083
Full time	310 (24.2)	238 (32.4)	124 (16.9)	44 (6.0)	19 (2.6)	
Part-time	117 (40.8)	85 (29.6)	58 (20.2)	17 (5.9)	10 (3.5)	
Homemaker	61 (40.7)	46 (30.7)	35 (23.3)	6 (7.3)	2 (1.3)	
Unemployed	100 (47.0)	64 (30.0)	35 (16.4)	10 (4.7)	4 (1.9)	
Retired	82 (58.2)	34 (24.1)	23 (16.3)	1 (0.7)	1 (0.7)	
Other/declined	55 (45.8)	36 (30.0)	20 (26.7)	4 (4.9)	5 (4.2)	
**Education**						0.702
High school or less	110 (44.5)	74 (30.0)	48 (19.4)	12 (4.9)	3 (1.2)	
Some college or technical school	102 (42.9)	71 (29.8)	46 (19.3)	12 (5.0)	7 (2.9)	
Bachelor's degree	271 (42.8)	198 (31.3)	121 (19.1)	31 (4.9)	12 (1.9)	
Master's degree or higher	231 (45.6)	155 (30.6)	77 (15.2)	26 (5.1)	18 (3.6)	
**Household Income ($)**						0.001
≤ 25,000	156 (55.1)	64 (22.6)	50 (17.7)	9 (3.2)	4 (1.4)	
> 25,000 –75,000	223 (42.1)	168 (31.7)	101 (19.1)	26 (4.9)	12 (2.3)	
>75,000 – 150,000	170 (41.7)	136 (33.3)	68 (16.7)	24 (5.9)	10 (2.5)	
> 150,000	111 (43)	86 (33.3)	41 (15.9)	17 (6.6)	3 (1.2)	
Decline to state	65 (38.9)	49 (29.3)	35 (21.0)	6 (3.6)	12 (7.2)	
**Concerns about the Vaccine**						<0.001
None	320 (82.9)	49 (12.7)	12 (3.1)	2 (0.5)	3 (0.8)	
Any	405 (32.1)	454 (36.0)	283 (22.5)	80 (6.4)	38 (3.0)	
**Length of SIP**^2^**order**	0.145					
No order	37 (39.8)	29 (31.2)	15 (16.1)	10 (10.8)	2 (2.2)	
< 1 month	39 (35.1)	46 (41.4)	18 (16.2)	4 (3.6)	4 (3.6)	
1 to < 2 months	103 (45.4)	69 (30.4)	36 (15.9)	12 (5.3)	7 (3.1)	
2 to < 3 months	103 (46.2)	74 (33.2)	30 (13.5)	13 (5.8)	3 (1.4)	
3 months or longer	383 (44.8)	247 (28.9)	163 (19.1)	39 (4.6)	23 (2.7)	
Don't know	57 (43.2)	37 (28.0)	32 (24.2)	4 (3.0)	2 (1.5)	
**The severity of COVID where you live**	0.019					
A lot less	73 (50.0)	28 (19.2)	31 (21.2)	8 (5.5)	6 (4.1)	
Somewhat less	163 (48.2)	97 (28.7)	57 (16.9)	12 (3.6)	9 (2.7)	
About the same	168 (40.3)	152 (36.5)	72 (17.3)	18 (4.3)	7 (1.7)	
Somewhat more	220 (43.8)	161 (32.1)	86 (17.1)	25 (5.0)	10 (2.0)	
A lot more	100 (42.4)	61 (25.9)	48 (20.3)	19 (8.1)	8 (3.4)	
**COVID-19 Effect on family income/employment**	0.122					
No change	263 (44.8)	170 (29.0)	103 (17.6)	34 (5.8)	17 (2.9)	
Mild	228 (41.2)	192 (34.7)	102 (18.4)	23 (4.2)	9 (1.6)	
Moderate	208 (47.4)	125 (28.5)	76 (17.3)	20 (4.6)	10 (2.3)	
Severe	23 (41.1)	14 (25.0)	10 (17.9)	5 (8.9)	4 (7.1)	
**Census Region**	0.116					
Midwest	37 (41.1)	35 (38.9)	15 (16.7)	1 (1.1)	2 (2.2)	
Northeast	55 (38.7)	45 (31.7)	31 (21.8)	7 (4.9)	4 (2.8)	
South	120 (39.3)	98 (32.1)	54 (17.7)	25 (8.2)	8 (2.6)	
West	513 (46.3)	325 (29.3)	195 (17.6)	49 (4.4)	27 (2.4)	
**Self-reported Health, quintiles**						0.411
Quintile 1 (lowest)	154 (44.6)	106 (30.7)	58 (16.8)	17 (4.9)	10 (2.9)	
Quintile 2	122 (46.2)	74 (28.0)	53 (20.1)	10 (3.8)	5 (1.9)	
Quintile 3	149 (43.5)	114 (35.1)	53 (16.4)	13 (4.0)	3 (0.9)	
Quintile 4	150 (42.7)	100 (28.5)	72 (20.5)	17 (4.8)	12 (3.4)	
Quintile 5 (highest)	119 (44.1)	86 (31.9)	39 (14.4)	18 (6.7)	8 (3.0)	

1 LEP: Limited English Proficiency

2 SIP: Shelter-in-Place

### 3.4 Multivariate analyses

**Fig. 1:**
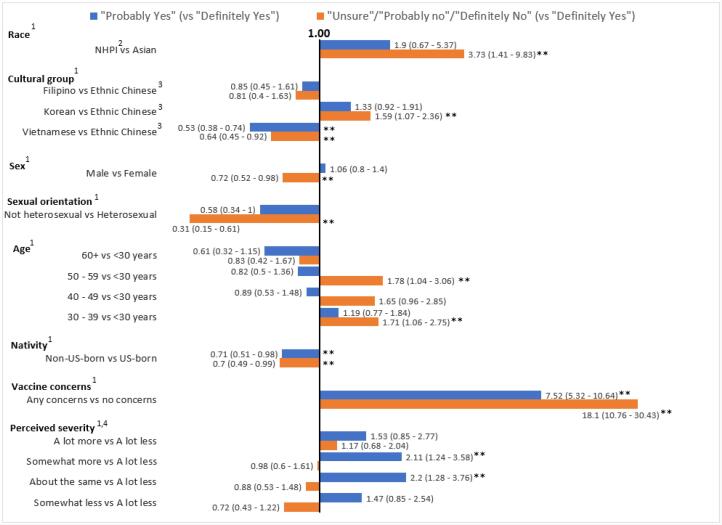
**Adjusted Odds Ratios and 95% Confidence Intervals for Willingness to be Vaccinated (N = 1,646)** (Centers for Disease Control and Prevention, 2021) Reference groups for independent variables: Race- Asian; Cultural group- Ethnic Chinese; Sex- Female; Sexual orientation- Heterosexual; Age- < 30 years; Nativity- US-born; Vaccine concerns- No concerns; Perceived severity- A lot less. (Food and Administration, 2021) NHPI: Native Hawaiian and Pacific Islanders. Ethnic Chinese includes mainland Chinese, Hongkonger, Taiwanese, and Huaren. (Food and Administration, 2021) Perceived severity: How would you rate the severity of COVID-19 outbreak at where you live in comparison to other locations in the US? ** *p* < 0.05.

The adjusted multinomial models are shown in Table 3 and depicted in Fig. 1. NHPI were significantly more likely to be “unsure/probably no/definitely no” versus “definitely yes” about getting the vaccine (vs. Asian Americans) (adjusted Odds Ratio (aOR), 3.73 [95% CI, 1.41–9.83], *P* < 0.01).

**Table 3:** Crude and Adjusted^1^ Odds Ratios (ORs) and 95% Confidence Intervals (CIs) for Willingness to be Vaccinated.

		Crude OR		Adjusted OR^1^	
	N	Probably Yes	Unsure/ Probably No/Definitely No	Probably Yes	Unsure/Probably No/Definitely No
**Race**					
Asian	1607	Reference	Reference	Reference	Reference
NHPI^2^	39	1.45 (0.60–3.51)	3.41 (1.57–7.39)	1.90 (0.67–5.37)	**3.73 (1.41 – 9.83)**
**Cultural Group**					
Ethnic Chinese^3^	524	Reference	Reference	Reference	Reference
Filipino		1.06 (0.59–1.87)	1.16 (0.62–2.16)	0.85 (0.45–1.61)	0.81 (0.40–1.63)
Korean	337	1.20 (0.88–1.64)	**1.49 (1.07**–**2.09)**	1.33 (0.92–1.91)	**1.59 (1.08**–**2.36)**
Vietnamese	478	**0.44 (0.33**–**0.59)**	**0.60 (0.44**–**0.81)**	**0.53 (0.38**–**0.74)**	**0.64 (0.45**–**0.92)**
Other/Mixed	307	0.65 (0.42–1.01)	1.22 (0.80–1.87)	**0.58 (0.35**–**0.98)**	1.00 (0.59–1.69)
**Sex**					
Female	1028	Reference	Reference	Reference	Reference
Male	601	0.96 (0.76–1.21)	0.60 (0.46–0.78)	1.06 (0.80–1.40)	**0.72 (0.52**–**0.98)**
**Sexual Orientation**					
Heterosexual	1478	Reference	Reference	Reference	Reference
Non-Heterosexual	95	0.65 (0.4–1.06)	0.40 (0.21–0.73)	**0.58 (0.34 – 1.00)**	**0.31 (0.15**–**0.61)**
**Age, years**					
< 30	535	Reference	Reference	Reference	Reference
30 – 39	339	**1.44 (1.05**–**1.98)**	**1.52 (1.08**–**2.15)**	1.19 (0.77–1.84)	**1.71 (1.06**–**2.75)**
40 – 49	237	0.97 (0.68–1.40)	1.39 (0.95–2.02)	0.89 (0.53–1.48)	1.65 (0.96–2.85)
50 – 59	295	0.91 (0.65–1.28)	1.40 (0.98–1.98)	0.82 (0.50–1.36)	**1.78 (1.04**–**3.06)**
≥ 60	240	**0.52 (0.36**–**0.75)**	**0.57 (0.38**–**0.85)**	0.61 (0.32–1.15)	0.83 (0.42–1.67)
**Nativity**					
US born	1012	Reference	N/A^a^	Reference	Reference
Foreign born	619	0.69 (0.55–0.88)	0.81 (0.63–1.05)	**0.71 (0.51 – 0.99)**	**0.70 (0.49**–**0.99)**
**LEP**^4^**in speaking**					
Yes	335	0.83 (0.63–1.11)	0.97 (0.72–1.30)	N/A^1^	N/A^1^
No	1311	Reference	Reference		
**LEP**^4^**in reading**					
Yes	309	0.87 (0.65–1.16)	1.00 (0.73–1.35)	N/A^1^	N/A^1^
No	1337	Reference	Reference		
**LEP**^4^**in writing**					
Yes	337	0.95 (0.71–1.26)	1.05 (0.78–1.41)	N/A^1^	N/A^1^
No	1309	Reference	Reference		
**Marital Status**					
Single	564	0.95 (0.75–1.21)	0.99 (0.77 – 1.29)	0.88 (0.60 – 1.30)	1.23 (0.81 – 1.87)
Married/Living with partner	988	Reference	Reference	Reference	Reference
Separated/Divorced/Widowed	83	0.34 (0.18 – 0.63)	0.58 (0.33 – 1.01)	0.59 (0.29 – 1.18)	0.71 (0.36 – 1.37)
**Employment Status**					
Full time	735	Reference	Reference	Reference	Reference
Part-time	287	0.95 (0.68 – 1.31)	1.20 (0.86 – 1.68)	1.19 (0.81 – 1.75)	1.30 (0.87 – 1.96)
Homemaker	150	0.98 (0.65 – 1.49)	1.17 (0.76 – 1.80)	1.05 (0.63 – 1.75)	0.96 (0.57 – 1.64)
Unemployed	213	0.83 (0.58 – 1.19)	0.81 (0.55 – 1.20)	1.09 (0.71 – 1.67)	0.92 (0.58 – 1.46)
Retired	141	0.54 (0.35 – 0.83)	0.50 (0.31 – 0.82)	1.14 (0.58 – 2.21)	1.08 (0.53 – 2.23)
Other/declined	120	0.85 (0.54 – 1.34)	0.87 (0.54 – 1.42)	0.98 (0.57 – 1.67)	0.92 (0.51 – 1.66)
**Education**					
≤ High school	247	Reference	Reference	N/A^1^	N/A^1^
Some college or technical	238	1.04 (0.68–1.58)	1.11 (0.72–1.73)		
Bachelor’s degree	633	1.09 (0.77–1.54)	1.06 (0.73–1.52)		
≥ Master’s degree	507	1.00 (0.70–1.43)	0.92 (0.63–1.34)		
**Household Income ($)**					
≤ 25,000	283	Reference	Reference	Reference	Reference
> 25,000 –75,000	530	**1.84 (1.29**–**2.61)**	**1.54 (1.08**–**2.22)**	1.24 (0.82–1.87)	1.10 (0.71 – 1.71)
>75,000 – 150,000	408	**1.95 (1.35**–**2.82)**	**1.49 (1.01**–**2.18)**	1.16 (0.75–1.78)	0.94 (0.60–1.49)
> 150,000 +	127	**1.89 (1.26**–**2.83)**	1.36 (0.89–2.09)	1.19(0.72–1.97)	0.87 (0.50–1.49)
**Concerns about the Vaccine**					
None	386	Reference	Reference	Reference	Reference
Any	1260	**7.32 (5.27**–**10.17)**	**18.64 (11.23**–**30.94)**	**7.52 (5.32**–**10.64)**	**18.10 (10.76**–**30.43)**
**Length of SIP**^5^**Order**					
No order	93	Reference	Reference	N/A^1^	N/A^1^
< 1 month	111	1.51 (0.79–2.87)	0.91 (0.45–1.84)		
1 - < 2 months	227	0.86 (0.48–1.52)	0.73 (0.40–1.33)		
2 - < 3 months	223	0.92 (0.52–1.62)	0.61 (0.33–1.12)		
≥ 3 months	855	0.82 (0.49–1.37)	0.81 (0.48–1.36)		
**Severity of COVID-19**					
A lot less	146	Reference	Reference	Reference	N/A^1^
Somewhat less	338	1.55 (0.94–2.57)	0.78 (0.49–1.23)	1.47 (0.85–2.54)	0.72 (0.43–1.22)
About the same	417	**2.36 (1.45**–**3.84)**	0.94 (0.60–1.47)	**2.20 (1.28**–**3.76)**	0.88 (0.53–1.48)
Somewhat more	502	**1.91 (1.18**–**3.09)**	0.89 (0.58–1.38)	**2.11 (1.24**–**3.58)**	0.98 (0.60–1.61)
A lot more	236	1.59 (0.93–2.73)	1.22 (0.76–1.96)	1.53 (0.85–2.77)	1.17 (0.68–2.04)
**COVID-19 Effect on Family Income/employment**					
No change	587	Reference	Reference	N/A^1^	N/A^1^
Mild	554	1.30 (0.99–1.71)	1.00 (0.75–1.34)		
Moderate	439	0.93 (0.69–1.25)	0.87 (0.64–1.18)		
Severe	56	0.94 (0.47–1.88)	1.41 (0.75–2.67)		
**Census Region**					
Midwest	90	1.49 (0.92–2.42)	0.92 (0.51–1.65)	N/A^1^	N/A^1^
Northeast	142	1.29 (0.85–1.96)	1.45 (0.94–2.22)		
South	305	1.29 (0.95–1.74)	1.37 (1.00–1.88)		
West	1109	Reference	Reference		
**Self-reported Health, quintiles**					
Quintile 1 (lowest)	345	Reference	Reference	N/A^1^	N/A^1^
Quintile 2	264	0.88 (0.60 – 1.29)	1.01 (0.68–1.50)		
Quintile 3	324	1.18 (0.83 – 1.67)	0.89 (0.60 – 1.31)		
Quintile 4	351	0.97 (0.68 – 1.38)	1.22 (0.85 – 1.76)		
Quintile 5 (highest)	270	1.05 (0.72 – 1.52)	0.99 (0.67 – 1.48)		

1 Adjusted for age, sex, race, cultural group, sexual orientation, nativity, COVID-19 severity, marital status, employment status, vaccine concerns

2 NHPI: Native Hawaiian and Pacific Islanders

3 Ethnic Chinese includes mainland Chinese, Hongkonger, Taiwanese, and Huaren.

4 English proficiency categorized as limited if speak/read/write English indicated as “some”, “a little” or “not at all”

5 SIP: Shelter-in-Place

Compared to Chinese, the adjusted OR for Koreans being “unsure/probably no/definitely no” versus “definitely yes” was 1.59 [95% CI, 1.08–2.36], *P* = 0.02 and for Vietnamese was 0.64 [95% CI, 0.45–0.92], *P* < 0.01. There were no statistically significant differences between Filipinos and Chinese. In post-hoc testing, compared to Koreans, Vietnamese were significantly less likely to be “unsure/probably no/definitely no” (aOR, 0.40 [95% CI, 0.27–0.62], *P* < 0.01 and “probably yes” about taking the vaccine, versus “definitely yes” (aOR, 0.40 [95% CI, 0.27–0.59], *P* < 0.01). There were no statistically significant differences between Filipinos and Vietnamese or Koreans (data not shown).

Sex and sexual orientation remained significantly associated with vaccine willingness in the adjusted model, with males (vs. females) and non-heterosexuals (vs. heterosexuals) less likely to be “unsure/probably no/definitely no” versus “definitely yes”.

Compared to those aged < 30 years, those aged 30–39 years were significantly more likely to state they were “unsure/probably no/definitely no” about getting the vaccine compared to those who said “definitely yes” (aOR,1.71 [95% CI, 1.06–2.75], *P* = 0.03). Those aged 30–39 years were significantly more likely to report “probably yes” about getting the vaccine compared to “definitely yes” (aOR, 1.44 [95% CI, 1.05–1.98], *P* < 0.01). Those aged 50–59 years were significantly more likely than those < 30 years to state they were “unsure/probably no/definitely no” about getting the vaccine (aOR, 1.78 [95% CI, 1.04–3.06], *P* = 0.04) compared to “definitely yes”.

Vaccine concerns (had concerns vs. not) were significantly associated with vaccine willingness: those who reported “unsure/probably no/definitely no” versus “definitely yes” (aOR, 18.10 [95% CI, 10.76–30.43], *P* < 0.01), and for those who reported “probably yes” versus “definitely yes” (aOR, 7.52 [95% CI, 5.32–10.64], *P* < 0 0.01).

## 4 Discussion

This paper reports willingness to receive the vaccine and factors related to willingness among 1,646 AAPI from a national multi-lingual survey before the safety and efficacy data were widely known, thus future research may investigate the potential changes in attitudes and factors associated with vaccination willingness. Results highlight the heterogeneity in vaccine willingness for AAPI. The study sought to provide these data to inform public health officials, community partners, and health care systems of potential outreach targets to improve vaccine willingness and thereby provide a defense against COVID-19 that has disproportionately affected AAPI. (Rubin-Miller et al., 2021, Chu et al., 2021)

From a health equity lens, the study provides disaggregated data for AAPI, which is significant because such data are scarce. These findings are among the first to reveal that AAPI groups are heterogeneous in COVID-19 vaccine willingness. This study found that NHPI were significantly less willing to receive the vaccine (vs. Asian Americans). This finding is critical as several U.S. states with large numbers of NHPI report higher rates of COVID-19 infections (vs. other racial/ethnic groups). (Kaholokula et al., 2020)Low vaccine willingness and high infection rates highlight the need for concentrated efforts to address COVID-19 health inequities for NHPI. The study also found that Korean Americans were less willing to receive the COVID-19 vaccine (vs. both Chinese and Vietnamese Americans), consistent with a global online survey that found that 25% of South Koreans (vs. 20% of respondents from China) indicated little/low vaccine intention. (Ipsos. Global attitudes on a COVID-19 vaccine: Ipsos survey for The World Economic Forum., 2021)The study found that Vietnamese Americans were more willing to receive the COVID-19 vaccine (vs. Chinese Americans), and such differences could not be explained by vaccine concerns or other correlates examined in this study. Results suggest targeted efforts for outreach to NHPI and Korean Americans, who were less willing to get the COVID-19 vaccine. Outreach in these languages and in areas where NHPI and Korean Americans are located may help to increase vaccine uptake. Public health campaigns with spokespersons from these cultural backgrounds may also help. Providers serving NHPI, Korean Americans, women and age groups that were less willing to receive the vaccine, could educate their patients about the importance of COVID-19 vaccination.

Overall, 25.4% of participants reported that they were unsure/unlikely to receive the vaccine, which closely mirrors the proportion (27%) reported in a national poll. (Kaiser Family Foundation, 2020)This study also found that those aged 30–39 (vs. < 30) were significantly less likely to be willing to get the vaccine, which is consistent with prior studies that found that being younger in age (Fisher et al., 2020) (e.g., 30–49) (Kaiser Family Foundation, 2020)was associated with vaccine hesitancy. However, the anomaly study finding was that those 50–59 (vs. aged < 30) were also less likely to be willing to get the vaccine; future research may investigate this association. Similar to prior findings, (Fisher et al., 2020, Callaghan et al., 2020, Kaiser Family Foundation, 2020, Reiter et al., 2020)those who reported having any vaccine concerns (vs. none) were less willing to receive the vaccine.

Consistent with another survey, this study found that men are more willing to get the vaccine than women. (Callaghan et al., 2020)This study also found that non-heterosexual AAPI were less likely to state that they were unwilling to get the vaccine (vs. heterosexual). Lesbian, gay, bisexual, transgender, queer, and intersex (LGBTQI + ) individuals experience health inequities that have become heightened with the pandemic. Reasons for greater vaccine willingness may be related to historical experiences of discrimination and health inequity such as lack of health insurance, (Kline, 2020)which may motivate individuals in sexual minority groups to access vaccination to reduce their risk for COVID-related morbidity/mortality. Greater vaccine willingness may also be related to the increased risk of infection that LGBTQI + individuals perceive with lower self-reported health status, higher rates of substance use, higher rates of HIV/AIDS (and compromised immune systems), and worse mental health (vs. general population). (Kline, 2020, Cancer, 2020, Pedrosa et al., 2020, Salerno et al., Aug 2020)Furthermore, in a poll of 174 adults who identified as LGBT, 75% viewed vaccination as a responsibility to protect others (vs. 48% of non-LGBT individuals). (Kaiser Family Foundation. The Impact of the COVID-19 Pandemic on LGBT People., 2021)

It is possible that participants who indicated that they were “unsure/probably no/definitely no” about getting the vaccine were waiting to receive additional information that could alleviate their concerns; this group can be considered the “wait and see” group. (National Academies of Sciences, 2020)A potential model that may be used as a guide to increase COVID-19 vaccine willingness is the World Health Organization BeSD Increasing Vaccination Model, (National Academies of Sciences, 2020)which stipulates “what people think and feel” (e.g., perceived risk) and the “social processes” (e.g., provider recommendations; social norms) lead up to the core of the model, “motivation” (e.g., willingness; hesitancy). By addressing “motivation” and barriers (e.g., cost; availability), individuals may be more likely to get the COVID-19 vaccine. (January, 2021)Culturally appropriate/tailored strategies are likely to play pivotal roles in vaccine uptake. For example, faith-based organizations may be important community partners for COVID-19 vaccine uptake, as they have been for other health promotion activities such hypertension prevention. (Kwon et al., 2017)Removing access barriers will be essential for the vaccine rollout. By providing multilingual helpline and resources and being geographically relevant (i.e., taking services to neighborhoods with higher density of Asian Americans), Asian Health Services (a Federally Qualified Health Center in the San Francisco Bay Area) saw an increase in both COVID-19 testing and vaccinations among Asian American individuals. (Quach et al., 2021)On a national scale, National Asian Pacific Center on Aging (NAPCA, a community partner of COMPASS) operates a helpline available in 6 AAPI languages to provide information and support with booking vaccination appointments. (Center, 2021)Additionally, recommendations from research for other types of vaccine uptake (e.g., HPV vaccine among Asian Americans) may be useful to apply in COVID-19 vaccine intention/uptake for AAPI (e.g., culturally appropriate educational information; family involvement). (Vu et al., 2020)

## 5 Limitations

There are a few limitations. First, this study is not an etiological study; however, future work may include repeated measures to assess change over time. Second, due to feasibility, not all AAPI languages were available, thus, limiting the ability to generalize findings to all AAPI. Thirdthe online survey limits the potential reach to AAPI with limited access to digital technology; however, COMPASS does offer toll-free numbers in the target languages. Fourth, the sample size for certain AAPI groups (e.g., NHPI; Asian Indians) were small; COMPASS’ present targeted outreach with NHPI and Asian Indian community partners may help to improve this. A strength of COMPASS is that community partners were engaged to help in the outreach/recruitment, and administration, which research has shown to be critical to successful AAPI. (Rabinowitz and Gallagher-Thompson, 2010, Grill and Galvin, 2014)

## 6 Conclusions

Strong willingness to get the COVID-19 vaccine is vital for personal, public, economic, and social health. (National Academies of Sciences, 2020) More than 25% of the participants indicated an unwillingness to get the COVID-19 vaccine; however, unwillingness significantly differed across AAPI groups, which underscore the need for data disaggregation, as an understanding of vaccine unwillingness (among a variety of other important outcomes) may be obscured by lumping AAPI together. A multi-pronged approach in culturally appropriate and tailored health communication and education is critical to achieve the goal of health equity for AAPI especially given the high burden of COVID-19-related morbidity and mortality among AAPI. (Marcello et al., 2020)Additionally, COMPASS was a multi-lingual survey available in multiple mediums (online, phone, and limited in-person) that allowed for a diverse sample of AAPI. Future research is encouraged to include non-English languages as well to help diversify their samples.

## 7 Role of the Funder/Sponsor

The funding source has no role in the design and conduct of the study; collection, management, analysis, and interpretation of the data; preparation, review, or approval of the manuscript; or decision to submit the manuscript for publication.

**Data Sharing Statement**

We will follow the NIH data sharing policy, https://grants.nih.gov/grants/policy/data_sharing/.

## Funding

This study was supported by a COVID-19 administrative supplement grant from the National Institutes of Health/National Institute on Aging (NIH/NIA) (3R24AG063718-02S1). Part of < named co-author > effort was supported by NIH/NIA R01AG067541.

## CRediT authorship contribution statement

**Van M. Ta Park:** Conceptualization, Methodology, Supervision, Investigation, Project administration, Funding acquisition, Writing - original draft, Writing - review & editing. **Marcelle Dougan:** Formal analysis, Visualization, Writing - original draft, Writing - review & editing. **Oanh L. Meyer:** Project administration, Writing - original draft, Writing - review & editing. **Bora Nam:** Project administration, Visualization, Writing - original draft, Writing - review & editing. **Marian Tzuang:** Project administration, Writing - original draft, Writing - review & editing. **Linda G. Park:** Writing - original draft, Writing - review & editing. **Quyen Vuong:** Project administration. **Janice Y. Tsoh:** Conceptualization, Methodology, Writing - original draft, Writing - review & editing.

## Declaration of Competing Interest

The authors declare that they have no known competing financial interests or personal relationships that could have appeared to influence the work reported in this paper.
